# Hydrogen Bonding with Hydridic Hydrogen–Experimental Low-Temperature IR and Computational Study: Is a Revised Definition of Hydrogen Bonding Appropriate?

**DOI:** 10.1021/jacs.3c00802

**Published:** 2023-04-10

**Authors:** Svatopluk Civiš, Maximilián Lamanec, Vladimír Špirko, Jiří Kubišta, Matej Špet’ko, Pavel Hobza

**Affiliations:** † Institute of Organic Chemistry and Biochemistry, Czech Academy of Sciences, Flemingovo Náměstí 542/2, 160 00 Prague, Czech Republic; ‡ J. Heyrovský Institute of Physical Chemistry, Czech Academy of Sciences, Dolejškova 2155/3, 18200 Prague 8, Czech Republic; § IT4Innovations, VŠB − Technical University of Ostrava, 17. listopadu 2172/15, 708 00 Ostrava-Poruba, Czech Republic; ∥ Department of Physical Chemistry, Palacký University Olomouc, tr. 17. listopadu 12, 771 46 Olomouc, Czech Republic

## Abstract

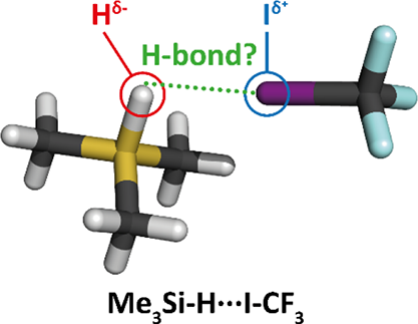

Spectroscopic characteristics of Me_3_Si–H···Y complexes (Y = ICF_3_, BrCN, and HCN) containing a hydridic hydrogen were determined experimentally by low-temperature IR experiments based on the direct spectral measurement of supersonically expanded intermediates on a cold substrate or by the technique of argon-matrix isolation as well as computationally at harmonic and one-dimensional anharmonic levels. The computations were based on DFT-D, MP2, MP2-F12, and CCSD(T)-F12 levels using various extended AO basis sets. The formation of all complexes related to the redshift of the Si–H stretching frequency upon complex formation was accompanied by an increase in its intensity. Similar results were obtained for another 10 electron acceptors of different types, positive σ-, π-, and p-holes and cations. The formation of HBe–H···Y complexes, studied only computationally and again containing a hydridic hydrogen, was characterized by the blueshift of the Be–H stretching frequency upon complexation accompanied by an increase in its intensity. The spectral shifts and stabilization energies obtained for all presently studied hydridic H-bonded complexes were comparable to those in protonic H-bonded complexes, which has prompted us to propose a modification of the existing IUPAC definition of H-bonding that covers, besides the classical protonic form, the non-classical hydridic and dihydrogen forms.

## 1 Introduction

Most of the elements in the periodic table have lower electronegativity than hydrogen (2.2), and only a few of them (C, N, O, F, S, Cl, Se, Br, and I) have it higher. Covalent bonds of hydrogen with the more electronegative atom X are characterized by the polarization of the X–H bond and the formation of a partial positive charge on hydrogen (protonic hydrogen). The molecule thus acts as a Lewis acid, and when it interacts with the electron donor Y (Lewis base), a hydrogen bond (H-bond) X–H···Y is formed.^1^ H-bonds are among the strongest types of non-covalent interactions and are the most common. The specificity of H-bonding is related to its easily detectable spectroscopic manifestation, which originates in different masses of H and X atoms. The formation of the X–H···Y H-bond, accompanied by a charge transfer from the Lewis base to the Lewis acid, results in a significant change of the X–H covalent bond. NBO orbital analysis^2^ has revealed a charge transfer from the lone pair of Y to the X–H σ* antibonding orbital. The increase in electron density in the σ* antibonding orbital weakens the X–H covalent bond and lowers the X–H stretching frequency (redshift).^3^ Our theoretical work at the end of the last century^4^ showed that the formation of the hydrogen bond could also be accompanied by a blueshift of the X–H stretching frequency. When our predictions were proven experimentally,^5^ it was obvious that there was a new type of H-bonding, for which we suggested a new name—blueshifting H-bonding. Extensive discussion in the computational and spectroscopic community led to the proposal of a new definition covering both types of H-bonding. The new IUPAC definition^6^ of the X–H···Y hydrogen bond thus includes both the weakening and strengthening of the X–H covalent bond, leading to red- and blueshifts of the X–H stretching frequency. Nevertheless, the characterization of atom X (X is defined as more electronegative than hydrogen) remained the same. As shown above, however, most of the elements in the periodic table are less electronegative (more electropositive). In these complexes, the interaction scheme is the same, namely, X–H···Y, but the hydrogen carries a negative charge (hydridic hydrogen) and the molecule acts as a Lewis base, whereas the Y atom carries a positive charge and the molecule acts as a Lewis acid. Both interaction types can be schematically represented as protonic, X–H^δ+^···Y^δ−^, and hydridic, X–H^δ−^···Y^δ+^. It should be emphasized that a very important feature of hydrogen bonding that makes its detection easy, namely, the position of light hydrogen between two much heavier atoms, is retained. This leads to an important question: does the second scheme correspond to H-bonding or is it another interaction type with a different definition and name? Many complexes containing Lewis bases including Si–H, Ge–H, Be–H, Mg–H, Zn–H, Li–H, and Cu–H hydridic bonds and different Lewis acids have been studied computationally in the laboratory of Jabłoński, who has recommended using the name charge-inverted H-bond^7−15^ (CIHB). The experimental detection of these non-classical H-bonds is, however, missing; their very existence is thus based on solving the Schrödinger equation only within the rigid rotor–harmonic oscillator–ideal gas approximation. The only exception is one class of CIHB, so-called dihydrogen bonding, where hydridic hydrogen interacts with protonic hydrogen. The structures of several dihydrogen-bonded complexes have been determined by X-ray and neutron diffraction studies; for some of them, theoretical calculations have revealed spectral shifts upon dihydrogen-bond formation.^16^ Finally, clusters of phenol and aniline with borane–amines having the B–H···H–X dihydrogen bond have been studied in supersonic jets using electronic and vibrational spectroscopy.^17^

Noncovalent interactions have been studied using many different experimental approaches, starting from classical solution-phase studies involving spectroscopic and thermodynamic measurements. More recently, important, complementary information to that obtained in solutions has been provided by studies in the gas phase, particularly the supersonic beam studies of Flygare, Klemperer, and others.^18−21^ The binding energy of weakly bound complexes is usually much smaller than room-temperature thermal energy. For this reason, the low temperature of a gas-phase supersonic jet, a rare-gas solid matrix, or a liquid helium droplet is the typical temperature in which noncovalent complexes are studied in the laboratory.

Besides the direct high-resolution gas-phase techniques, there are also other very powerful low-temperature methods. The possibility of storing spectroscopically detectable concentrations of reaction intermediates in a solid or rare-gas matrix was first recognized by Whittle, Dows, and Pimentel.^22^ These matrix materials are often chemically inert and are optically transparent from the far-infrared range well into the vacuum–ultraviolet region. The early experiments demonstrated that at 20 K, which is a temperature conveniently obtained using liquid hydrogen, solid nitrogen and argon are rigid enough to eliminate molecular diffusion and effectively inhibit subsequent chemical reactions. At the cryogenic temperatures required for studying rare-gas solids, molecules reside in their ground electronic and vibrational states. Since diffusion is inhibited, reaction intermediates do not undergo further reaction, and sufficient concentrations of many of them have been obtained for studies of their electronic and infrared spectra.

The aim of the present paper is to study the X′–H···Y′ complexes (X′ is more electropositive than hydrogen, X′–H acts as a Lewis base) containing Si–H and Be–H hydridic bonds and various hydridic–hydrogen acceptor Ys (Lewis acids) having a positive σ-, π-, and n-hole or a positive atom (e.g., hydrogen). Spectroscopic characteristics of the Me_3_Si–H···Y′ (Y′ = ICF_3_, BrCN and HCN) complexes obtained not only at the harmonic but also at the more reliable anharmonic level have been verified by low-temperature IR experiments. Notice that the present experiments are the first ones to confirm unambiguously the formation of the CIHB. Finally, an attempt has been made to find a new definition of H-bonding that would cover both protonic (red- as well as blueshifting) and hydridic (CIHB) schemes.

## 2 Instrumentation and Spectral Measurement

In our low-temperature experiments, two different approaches have been applied (for more details, see the SI).

The first of them (A) is the technique of the direct spectral measurement of a supersonically expanded mixture of reaction intermediates on a cold substrate (solid-phase complex, SPC) and the second (B) is the technique of noble-gas matrix isolation (MI).

(A) The procedure of low-temperature experiments has already been described elsewhere.^23,24^ The gas mixtures are usually deposited onto a cooled KBr substrate of the cryostat at a temperature of 4–20 K. The relative concentrations of the products have been monitored using the integrated absorption intensities of selected infrared bands. The intermediates forming part of the low-temperature complex have been supersonically expanded into a high vacuum (10^–6^ Torr) on a cold substrate (of 18 K, which is the minimum attainable temperature) inside a Leybold cryostat chamber. The spectra were obtained using a Bruker Vertex spectrometer with KBr optics, a HgCdTe detector, and a KBr beam splitter. The broad spectral region was cut by optical interference filters with transparency in the range of 700–5000 cm^–1^. The KBr entry window of the spectrometer was used. The unapodized spectral resolution was 0.06 cm^–1^. Between 30 and 100 scans, depending on the sample, were used to obtain a reasonable signal-to-noise ratio. The observed wavenumbers were calibrated using CO_2_-absorption rotation–vibration lines.

(B) Matrix isolation is a well-known technique frequently used for the measurement of unstable species such as ions, radicals, and low-temperature-existing molecular complexes in a cold matrix of noble gas (Ng). Like in the procedure A, a mixture of reaction intermediates mixed together with argon gas (molar ratio 1:1000) was expanded through a pulse nozzle onto the cold (18 K) KBr substrate, and the spectra were recorded using the Bruker Vertex spectrometer.

## 3 System Considered

**Figure 1:**
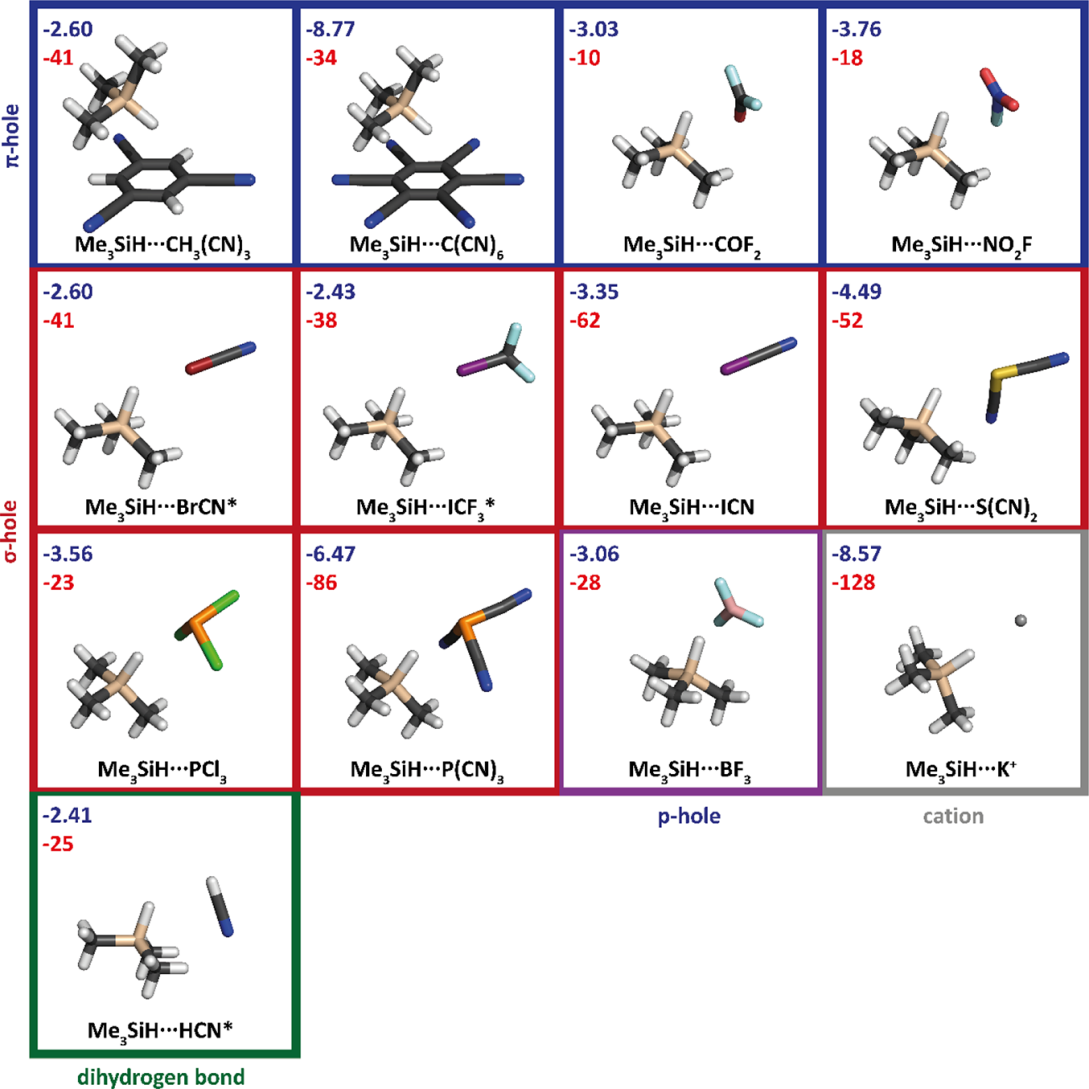
Geometries of the complexes investigated optimized at the MP2/cc-pwCVTZ level (cc-pwCVTZ-PP for Br and I): Si – beige, C – black, H – white, I −violet, F – light blue, Br – burgundy, N – dark blue, P – orange, Cl – green, S – yellow, O – red, K – silver, and B – rose. Dark blue is used for the total interaction energy (in kcal/mol) at the MP2/cc-pwCVTZ level for each complex, and red is used for the shift of the Si–H stretching frequency (in cm^–1^). The complexes with the experiment are marked with asterisks.

Me_3_Si–H and HBe–H systems containing a hydridic hydrogen and different electron acceptors (ICF_3_, BrCN, HCN, K^+^, C_6_(CN)_6_, C_6_H_3_(CN)_3_, BF_3_, P(CN)_3_, PCl_3_, S(CN)_2_, NO_2_F, CO_2_F, and ICN) forming CIHBs have been considered. Lewis bases contain a positive σ-, π-, and p-hole or a positive hydrogen. The latter complexes are also known as complexes with a dihydrogen bond. All complexes considered are depicted in Figure 1.

In this paper, we use both techniques A and B. It is well known that the method of matrix isolation gives narrow lines, rare-gas atoms isolate the molecules from mutual interaction, and subsequent chemical reactions are effectively inhibited. On the contrary, the A arrangement provides the possibility to study the basic energy characteristic of the molecular complexes, such as thermodynamic stability, on the board temperature scale.

## 4 Methods

### 4.1 Geometry Optimization and Thermodynamic Properties

Geometries of all subsystems and complexes were optimized at the RI-MP2/cc-pwCVTZ^25^ level. For heavy halogens (Br, I), basis sets with pseudopotentials (cc-pwCVTZ-PP^26^) were used. Thermodynamic properties as well as harmonic vibration frequencies, determined using the rigid rotor–harmonic oscillator–ideal gas approximation, were evaluated at the same theoretical level. Geometry optimization and harmonic vibrational analysis on selected complexes (ICF_3_, BrCn, and HCN complexes with Me_3_SiH) were also performed using the more reliable explicitly correlated RI-MP2-F12^27^ method in cc-pVTZ-F12^28^ basis sets (cc-pVTZ-PP-F12 for Br and I). All calculations were performed with MOLPRO 2022.^29,30^ The potential energy surfaces for the anharmonic calculations of Si–H frequencies were obtained on the MP2/cc-pVTZ level (cc-pwCVTZ-PP for Br and I) using the Cuby4^31^ framework and the TURBOMOLE 7.5^32^ program.

### 4.2 Single-Point Energy Calculations

CCSD(T)-F12^33^ energies were determined with cc-pVTZ-F12 (cc-pVTZ-PP-F12 for Br and I) basis sets implemented in the MOLPRO 2022 package on geometries obtained from the MP2–F12 method. All interaction energies were systematically corrected using the Boys and Bernardi counterpoise technique.^34^ Energy decomposition analysis was performed using the SAPT2 + 3^35^/aug-cc-pwCVTZ (aug-cc-pwCVTZ-PP for Br and I) method implemented in the PSI4 package.^36^

### 4.3 NBO Analysis

NBO analysis was performed at the ωB97X-D/cc-pwCVTZ level (cc-pwCVTZ-PP for Br and I) using the NBO^2^ program implemented in Gaussian 16.^37^

### 4.4 One-Dimensional Harmonic and Anharmonic Vibrational Analysis

Like in our previous publications,^38,39^ the evaluation of the sought vibrational Si–H stretching frequencies relies on the deep adiabatic separability of the Si–H mode (s) from the rest of the vibrational degrees of freedom of the probed compounds and on the use of a HBJ non-rigid reference configuration of the atomic nuclei that essentially follows the Si–H motion.^40^ The vibrational energies can then be obtained by solving the Schrödinger equation for the following Hamiltonian:
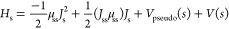
where *J*_s_ = – *iℏ*(*d*/*ds*), μ_ss_ is the Si–H stretching component of the tensor that is the inverse of the 4 × 4 generalized molecular inertia tensor, μ is the determinant of the matrix [μ_α,β_] (α,β = *x*, *y*, *z*, *s*), with *x*, *y*, and *z* being the Cartesian atomic coordinates in the molecule-fixed axis system, *V*_pseudo_(*s*) is a mass-dependent kinematic pseudopotential, and *V*(*s*) is the Si–H minimum-energy-path stretching potential.

Obviously, mainly for the practical impossibility to account reliably for the aggregation effects of the molecular environments used, the adopted “isolated-molecule” theory may seem inadequate. Nevertheless, if one assumes a purely linear dependence of the atomic coordinates of the probed compounds (deposited on cold substrates or trapped in rare-gas matrices) on the stretching Si–H distortion, the stretching-reduced mass μ_ss_ becomes constant and can thus be used as a single scaling parameter. Interestingly, as illustrated in Figure S1, the assumption of “linearity” seems to hold at least for the minimum-energy-path relaxation effects in isolated molecules (note that the *x* and *y* coordinates exhibit even much lower relaxation dependence than their *z*-counterparts). As shown in Figure S2, the “complete” scaling factors corresponding to the observed spectral shifts Δν obtained using the argon matrix-isolation technique acquire rather coinciding values, thus explicitly justifying the use of the “linearity” assumption for the rationalization of the Ar-matrix-isolation data. The anharmonic calculation thus appears to be a useful complement to the standard normal coordinate analysis.

## 5 Results and Discussion

### 5.1 Experiment

All complexes were studied in a solid phase on a cold (18 K) KBr substrate and simultaneously in the Ar matrix.

#### 5.1.1 Me_3_Si–H···ICF_3_ Complex

**Figure 2:**
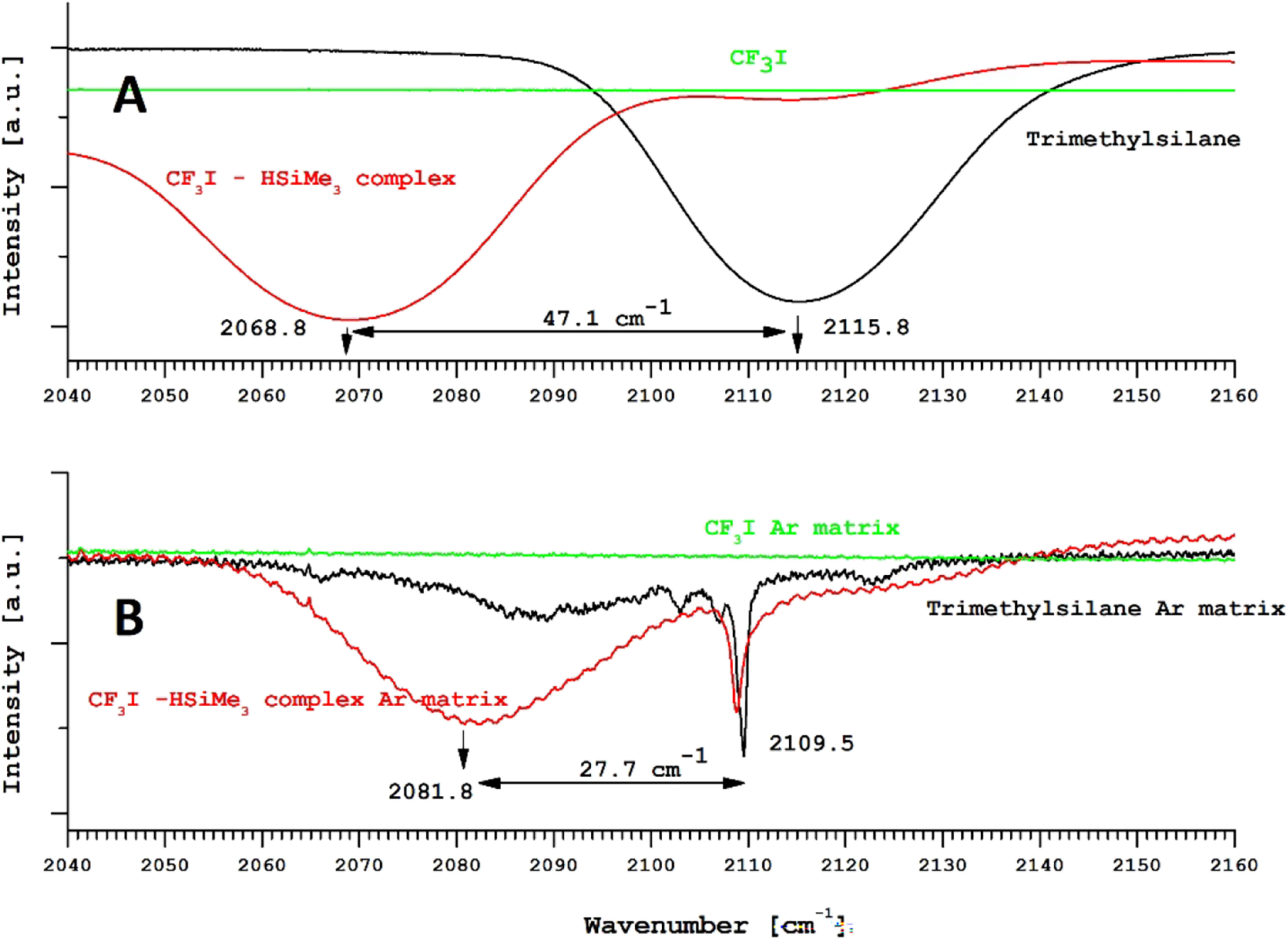
Solid-state and Ar-matrix spectra of the Me_3_Si–H···ICF_3_ complex.

Panel (A) of Figure 2 depicts the spectrum of trimethylsilane (black) on a cold (18 K) KBr substrate together with the spectrum of the trimethylsilane-CF_3_I 1:1 molecular complex (2068.8 cm^–1^) at 18 K. The obtained redshift of the Si–H bond was estimated to be about 47.1 cm^–1^. Panel (B) shows the Ar-matrix spectrum of pure trimethylsilane (black) and the spectrum of the Me_3_Si–H···CF_3_I complex at 18 K (red). The measured Si–H bond shift inside the argon matrix was 27.7 cm^–1^.

#### 5.1.2 Me_3_Si–H···BrCN Complex

**Figure 3:**
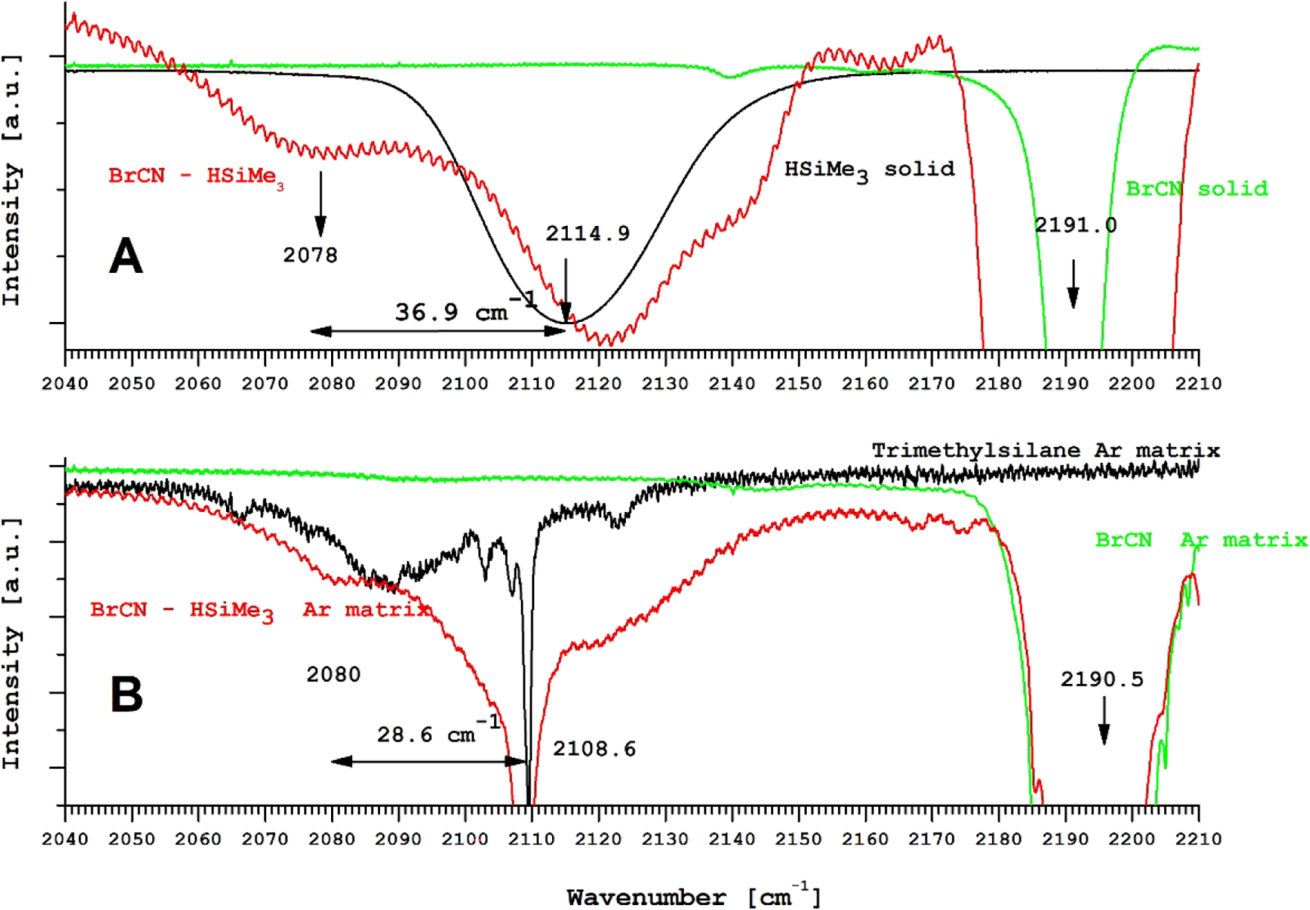
Solid-state and Ar-matrix spectra of the Me_3_Si–H···BrCN complex.

Panel (A) in Figure 3 shows the spectrum of trimethylsilane (black) on a cold (18 K) KBr substrate together with the absorption spectrum of BrCN at 18 K (green) and the Me_3_Si–H···BrCN–silane 1:1 molecular mixture at 18 K. If we accept the formation of the Me_3_Si–H···BrCN molecular complex (the broad peak around 2078 cm^–1^), the obtained redshift of the Si–H bond gives the value of 36.9 cm^–1^ (see Figure 3A). Panel (B) shows the Ar-matrix spectrum of pure trimethylsilane (black) and the spectrum of the Ar–Me_3_Si–H···BrCN mixture (2000,1) at 18 K (red) and the argon-matrix spectrum of BrCN (green). From the argon-matrix spectra (Figure 3B), we attributed the broad peak around 2080 cm^–1^ to the molecular Me_3_Si–H···BrCN complex, with the resulting redshift of the H-Br bond being 28.6 cm^–1^.

#### 5.1.3 Me_3_Si–H···HCN Complex

**Figure 4:**
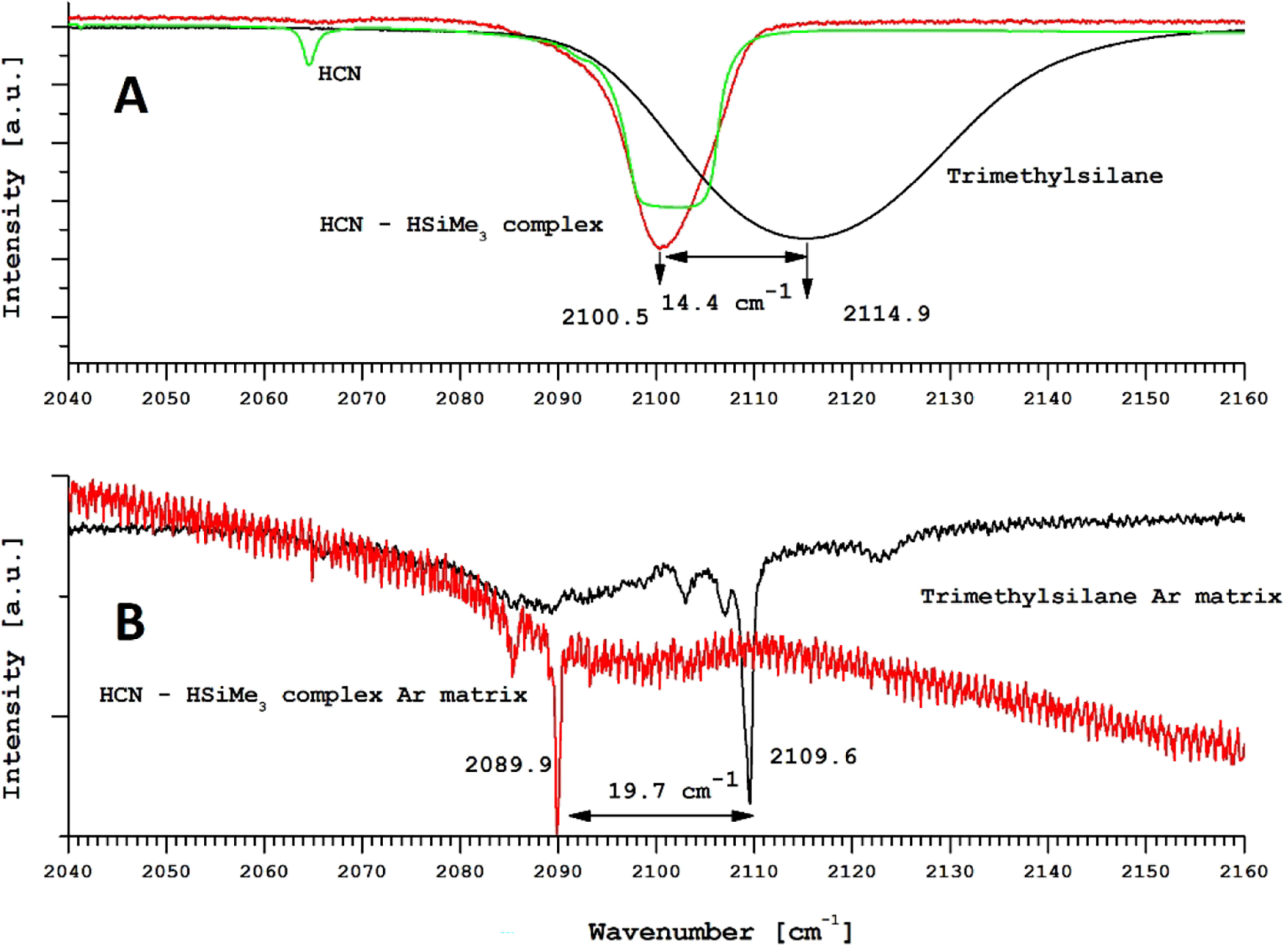
Solid-state and Ar-matrix spectra of the Me_3_Si–H···HCN complex.

Panel (A) in Figure 4 depicts the spectrum of trimethylsilane (black) on a cold (18 K) KBr substrate together with the HCN molecular absorption band at 18 K (green) and the Me_3_Si–H···HCN 1:1 molecular complex (2100.5 cm^–1^) at 18 K. The obtained redshift of the Si–H bond was estimated to be about 14.4 cm^–1^. Panel (B) shows the Ar-matrix spectrum of pure trimethylsilane (black) and the spectrum of the Me_3_Si–H···HCN complex at 18 K. The measured Si–H bond shift inside the argon matrix was 19.7 cm^–1^.

### 5.2 Calculations

#### 5.2.1 Subsystems

**Figure 5:**
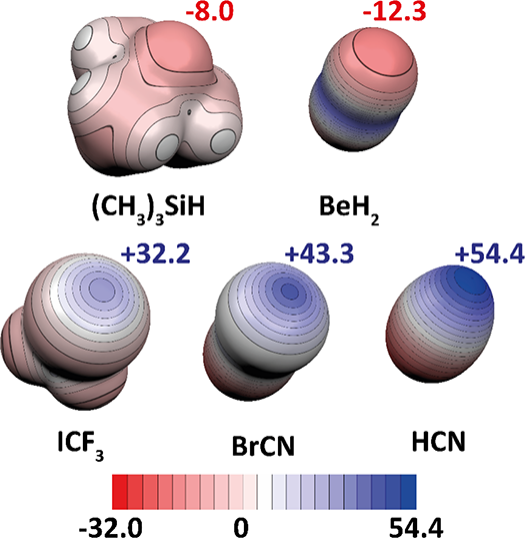
MEP calculated at the ωB97X-D/def2-QZVPP level for the monomers studied. The ESP scale is in kcal/mol. The *V*_s,max_ on the hydrogen atom from the Me_3_SiH molecule is depicted in red. The *V*_*s*,min_ in the center of the σ-hole or on top of the hydrogen atom is in blue.

Electrostatic potentials including the *V*_s,max_ and *V*_s,min_ values for the optimized structures of selected Lewis bases and acids (for which experimental measurements have been performed) are visualized in Figure 5. The most basic hydridic hydrogen (i.e., the strongest electron donor) has been found in BeH_2_, followed by Me_3_Si–H, while the strongest electron acceptor has been detected in HCN.

#### 5.2.2 Complexes

The intramolecular X′–H (X′–H) and intermolecular H...Y′ distances for Me_3_Si–H···Y′ complexes are shown in Table 1, whereas these characteristics for BeH_2_ complexes are summarized in Table S1. In comparison with the sum of the vdW radii,^41^ the intermolecular distances in complexes with ICF_3_ and BrCN are significantly shorter (by 0.573 and 0.585 Å); in the complex with HCN, it is longer. Table 1 further shows that all intramolecular Si–H distances are systematically elongated upon complex formation. Notice that the elongation of the Si–H bond is smaller than that of the X–H bonds in the X–H···Y H-bonded systems. As expected, the Si–C bonds are contracted, but the respective changes are smaller. Finally, upon complex formation, the I–C and Br–C intramolecular distances are elongated, whereas the H–C distance is not changed. Qualitatively similar results have also been obtained for HBeH···Y′ complexes.

**Table 1:** Intermolecular Distances and Changes in Intramolecular Bond Lengths (in Å) of Selected Bonds in Me_3_SiH···Y′ Complexes (Y′ = ICF_3_, BrCN, HCN) Calculated at the MP2-F12/cc-pVTZ-F12 Level^b^

	Δ*r*(Si–H)	Δ*r*(Si–C)	Δ*r*(I–C)	H···I/vdW^a^
ICF_3_	0.007	–0.003/–0.001/–0.001	0.002	2.874/–0.573

	Δ*r*(Si–H)	Δ*r*(Si–C)	Δ*r*(Br–C)	H···Br/vdW^a^
BrCN	0.008	–0.003/–0.002/–0.002	0.005	2.705/–0.585

	Δ*r*(Si–H)	Δ*r*(Si–C)	Δ*r*(H–C)	H···H/vdW^a^
HCN	0.005	–0.003/–0.003/0	0	2.729/+0.320

a The difference between the intermolecular distance and the sum of the vdW radii.

b All values are in Å.

#### 5.2.3 Energies

Table 2 contains calculated energy characteristics for Me_3_Si–H···Y′ complexes, specifically MP2, MP2–F12, and CCSD(T)-F12 interaction energies and binding free energies at 18 K based on the MP2 and MP2–F12 characteristics. Corresponding results obtained for BeH_2_ complexes are collected in Table S2.

**Table 2:** Energy Characteristics of the Me_3_SiH···Y′ Complexes (Y′ = ICF_3_, BrCN, HCN)^a^

Y′	Δ*E*^MP2^/Δ*E*^MP2-F12^ /Δ*E*^CCSD(T)-F12^	Δ*G*^MP2^(18 K)/Δ*G*^MP2-F12^(18 K)
ICF_3_	–2.43/–3.27/–2.70	–1.85/–2.42
BrCN	–2.60/–3.02/–2.76	–2.07/–2.77
HCN	–2.41/–2.59/–2.24	–1.79/–1.97

a The total interaction energy calculated at the MP2/cc-pwCVTZ level (Δ*E*^MP2^) and at the MP2-F12/cc-pVTZ-F12 level (Δ*E*^MP2-F12^), intrinsic interaction energy with counterpoise correction at the CCSD(T)-F12/cc-pVTZ-F12 level on the MP2-F12/cc-pVTZ-F12 geometries (Δ*E*^CCSD(T)-F12^) and Gibbs free energy at MP2/cc-pwCVTZ (Δ*G*^MP2^) and MP2-F12/cc-pVTZ-F12 (Δ*G*^MP2-F12^) levels. All values are in kcal/mol.

The MP2 stabilization energies of all CIHB complexes are comparable, whereas the more reliable MP2–F12 and mainly CCSD(T)–F12 energies of the first two complexes are similar and are higher than that of the third (dihydrogen-bonded) one. Further, all MP2–F12 and CCSD(T)–F12 stabilization energies lie between 2.6 and 3.3 kcal/mol and between 2.2 and 2.8 kcal/mol, respectively, and are thus well comparable to those of classical H-bonded complexes. These findings contradict the results from the previous subchapter (Subsystems), showing that the strongest electron acceptor is HCN, followed by BrCN and ICF_3_. Therefore, the complex with HCN was expected to be the strongest. The fact that the opposite is true, i.e., this complex is the weakest, is caused by dispersion energy. The SAPT2 + 3^35^ dispersion and total interaction energies for the complexes of Me_3_Si–H with CF_3_I, BrCN, and HCN amount to −4.35, −3.79, and −3.05 kcal/mol and – 3.34, −3.20, −2.50 kcal/mol, respectively. Clearly, the smallest dispersion energy for the last complex is responsible for its smallest stabilization energy. Notice that the SAPT2 + 3 interaction energies for all three complexes agree surprisingly well with the MP2–F12 ones. The negative binding free energies calculated for all the complexes indicate their formation at 18 K. The MP2 and CCSD(T) interaction energies of BeH_2_ complexes are systematically smaller, with the largest values found for the HBeH···BrCN complex. The binding free energies of all complexes are smaller than those of Me_3_Si–H, but they are still negative, which ensures their formation at 18 K.

Table 3 shows the difference in the orbital occupancies (of monomers and complexes) of occupied and unoccupied Si–H σ-orbitals (the corresponding values for the BeH_2_ complexes are included in Table S3). Changes in orbital occupancies are related to changes in bond lengths; the biggest changes have been found for the Si–H bond. A decrease in the occupancy of the Si–H σ-orbitals and its increase in the case of the σ*-orbitals, found for all three complexes, lead to a weakening of the Si–H bond, which is manifested by the elongation of the Si–H bond (cf. Table 1). Very similar results were obtained for the I–C, Br–C, and H–C bonds in the ICF3, BrCN, and HCN electron acceptors, respectively. In these cases, the elongation of the respective bonds was smaller and, in the case of HCN even, equal to zero.

**Table 3:** Orbital–Occupation Difference between Monomers and in Me_3_SiH···Y′ Complexes Using NBO Analysis Calculated at the ωB97X-D/aug-cc-pwCVTZ Level on MP2-F12/cc-pVTZ-F12 Geometries

Y′	σ/σ* Si–H	σ/σ* I–C	ΣLP I
ICF_3_	–0.010/0.004	–0.003/0.005	–0.005

Y′	σ/σ* Si–H	σ/σ* Br–C	ΣLP Br
BrCN	–0.010/0.004	–0.001/0.009	–0.002

Y′	σ/σ* Si–H	σ/σ* H–C	LP N
HCN	–0.001/0.002	–0.001/0	–0.001

Other occupancy changes and the respective bond-length changes are less pronounced and are not discussed below.

#### 5.2.4 Vibration Frequencies

Table 4 presents the experimental and calculated shifts of Si–H stretching frequencies upon complex formation. First, both experimental techniques predict larger shifts for the first two complexes; the same trend appears in all calculated shifts. Second, the calculated MP2–F12 harmonic shifts are systematically larger than the MP2 harmonic ones and are similar to the anharmonic unscaled ones. Finally, the agreement between the experimental and calculated frequencies is very good, which supports the reliability and suitability of all calculated techniques. In summary, the agreement between the experimental and calculated frequency shifts is satisfactory and all Si–H frequencies are redshifted upon complex formation. As expected, the magnitude of the redshifts correlates with the complex stability. Finally, the intensities of the Si–H stretch in all three complexes have increased upon complex formation. Notice here that the experimental technique adopted has not made it possible to detect intensity changes.

**Table 4:** IR Shift (Δν in cm^–1^) and Change of Intensity (Δ*I* in km/mol) of the Si–H Band upon the Formation of Me_3_SiH···Y′ Complexes

Y′	Δν^a^	ΔI^b^
ICF_3_	–47/–28//–31(−52)/–38(−49^c^)	+136
BrCN	–37/–29//–36(−55)/–41(−50)	+138
HCN	–14/–20//–20(−35)/–25(−29)	+37

a Experimental – cold substrate/experimental – Ar-matrix//calculated anharmonic scaled (calculated anharmonic unscaled)/calculated harmonic at the MP2/cc-pwCVTZ level (calculated harmonic at the MP2-F12/cc-pVTZ-F12 level).

b Calculated harmonic at the MP2/cc-pwCVTZ level.

c Geometry minimum with one negative frequency.

In the case of Be–H, the lower-energy symmetric stretching vibrations are forbidden; thus, only the antisymmetric vibrations, which are higher in energy, are discussed. As observed for the above-discussed Si–H frequencies, the intensities of the Be–H stretching frequencies (see Table S4 for the calculated harmonic Be–H stretching frequencies and their intensities) upon complexes formation also significantly increase. Frequency shifts, on the other hand, provide a different picture, with a blueshift in HBeH···BrCN, redshift in HBeH···HCN, and only negligible shift in HBeH···ICF_3_.

#### 5.2.5 Characterization and Classification of the Complexes Studied

**Figure 6:**
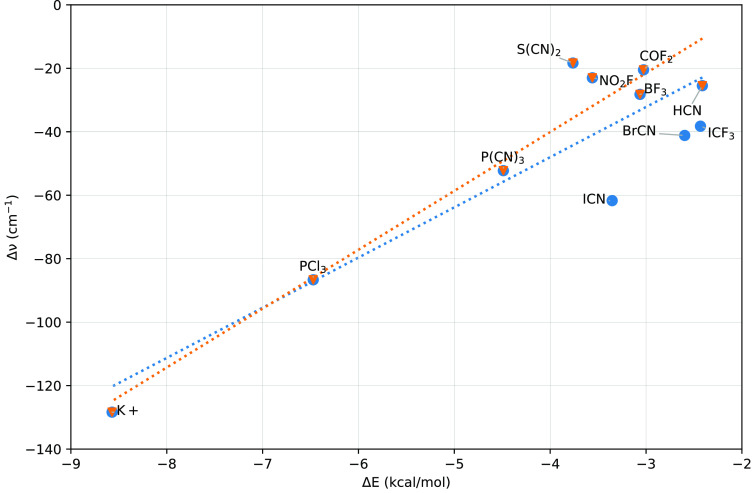
Correlation between the total interaction energy (Δ*E*) and the shift of the Si–H stretching frequency (Δν) for selected complexes of Me_3_SiH with various electron acceptors. All values are calculated at the MP2/cc-pwCVTZ level. The blue correlation includes all electron acceptors; in the orange one, the electron acceptors exhibiting σ-holes (ICN, BrCN, and ICF_3_) have been omitted.

The classification of Me_3_Si–H···Y′ (Y′ = ICF_3_, BrCN, HCN) complexes, studied both experimentally and computationally, is not unique. The first two complexes can be viewed either as CIHB or as halogen-bonded (XB) ones. Their classification is not easy because both forms are involved—the stabilization of the complex comes from Si–H···Y′ CIHB as well as from the H^δ-^ → I (Br) σ-hole halogen bond. The dominant role of the former contribution is supported by the following evidence:

(i) The CIHB is accompanied by a charge transfer (CT) from Y′ to Me_3_Si–H, whereas the CT accompanying the XB is reversed, i.e., from Me_3_Si–H to Y′. Because of the partial compensation of both contributions, the total CT between the subsystems should be small, which is fully supported by the calculated value (0.007 and 0.003 electrons transferred from Me3SiH to BrCN and ICF_3_, respectively). This is further verified by the NBO E2 charge-transfer energies (Me_3_Si–H → Y′ and Y′ → Me_3_Si–H), which are almost equal for both complexes. Table 3 shows that electron density in these two complexes is transferred predominantly to the σ- and σ* Si–H orbitals, which leads to the weakening and elongation of this bond, accompanied by a redshift of the Si–H stretching frequency. In the reverse case, the electron density in the X–C σ- and σ* orbitals of ICF_3_ and BrCN also decreases and increases, resulting again in the weakening and elongation of the bond accompanied by a redshift of the respective stretching frequencies. The redshifts of X–C stretching frequencies are smaller than that in that in the previous case (Si–H) due to the presence of two heavy atoms (I,C and Br,C). Shifts of the Si–H bond stretching vibration frequencies are thus the most visible presentation of the complex formation.
(ii) The strength of the XB in Me_3_Si–H···Y′ (Y′ = ICF_3_, BrCN) complexes can only be estimated indirectly. The stabilization energy of the XB complexes with identical electron acceptors and a different electron donor, namely, H_3_N···BrCN and H_3_N···ICF_3_, where stabilization comes exclusively from the halogen bond, is considerably higher (6.0 and 5.4 kcal/mol, respectively) than that of parent complexes with Me_3_Si–H (cf. Table 2). This finding corroborates the fact that XB in Me_3_Si–H···Y′ (Y′ = ICF_3_, BrCN) complexes is only weak.
(iii) The most conclusive evidence comes from the correlation between the stabilization energy and shift of the Si–H stretching frequency upon Me_3_Si–H···Y′ complex formation. Thirteen different electron acceptors have been considered here: first, those having pronounced σ- (ICF_3_, BrCN, ICN, P(CN)_3_, PCl_3_, S(CN)_2_,) and π- (CH_3_(CN)_3_, C(CN)_6_, COF_2_, NO_2_F) holes, allowing the formation of the XB; next, an electron acceptor with a p-hole (BF_3_); and, finally, electron acceptors with a positive charge (K^+^, HCN). The respective structures, stabilization energies, and shifts of the Si–H stretching frequencies are shown in Figure 1. The formation of CIHB is systematically manifested by the redshift of the Si–H stretching frequency. As expected, this shift is proportional to the stabilization energy of the complex (cf. Figure 6). The figure depicts two correlation lines: the blue one shows correlation for all electron acceptors, while in the case of the orange one, the electron acceptors exhibiting σ-holes (ICF3, BrCN, and ICN) have been omitted. Evidently, both correlations are very similar, which indicates that the nature of stabilization in the last three complexes is not significantly different from those complexes where XB character is absent.

Finally, the third complex for which experimental results exist, Me_3_Si–H...HCN, can be characterized as the CIHB or the dihydrogen one. In this case, the classification is easier because we can directly compare shifts of Si–H and C–H vibration stretching frequencies in both H-bonds as both possess hydrogen. Upon complex formation, the computed Si–H stretching frequency is redshifted (by 25 cm^–1^), whereas the C–H stretching frequency is blueshifted. The blueshift is, however, considerably smaller (7 cm^–1^). This means that the CIHB character of the complex is dominant over the dihydrogen-bonded one, which is further supported by correlation between the stabilization energy and vibration shifts, discussed in previous the paragraph (Me_3_Si–H···HCN is very close to the ideal correlation line).

We can thus conclude that in all three complexes studied experimentally as well as computationally, the CIHB form is dominant over the XB and dihydrogen forms. This is reflected in the moderate redshift of Si–H stretching frequency, which is the largest among the stretching frequencies. These shifts are well comparable to redshifts in complexes with protonic H-bonds.

## 6 Conclusions and Revised Definition of H-Bonding

The complexation of Me_3_Si–H and HBe–H molecules (containing a hydridic hydrogen) with different electron acceptors is accompanied by red- and blueshifts of the Si–H and Be–H stretching frequencies, which are comparable with those in protonic H-bonded complexes. Both the red- or blueshifts were accompanied by an increase in intensity (the experiment did not make it possible to detect the intensity changes during complex formation), comparable to intensity changes of protonic H-bonded complexes.

An important question arises as to what to call these interactions with hydridic–hydrogen participation. Jabłoński, who studied these interactions intensely, introduced a new name for them, namely, charge-inverted H-bonding. In our opinion, this name does not capture the nature of the interaction, and “hydridic H-bonding” seems more appropriate. The wide use of this term is, however, connected with another problem. If we characterize the type of hydrogen bond by the charge on the participating hydrogen, we should consistently use “protonic H-bond” instead of “H-bond”. The undesirable inflation of new names in this case can easily be avoided by changing the current IUPAC definition of hydrogen bonding. The current IUPAC definition requires “X (from X–H) to be more electronegative than H.” We prefer this avenue because the relevant change in the definition (mainly concerning the relative electronegativities of X and H) is only marginal and, besides hydridic and protonic H-bonding, it will also cover the dihydrogen bond. Below, we present the very first draft of this modification including a part of the original IUPAC definition, with the proposed corrections in italics and with the part of the definition to be removed underscored. The revised version covers three different interaction schemes where hydrogen plays a dominant role: X–H^δ+^···Y^δ−^ (protonic H-bond), X′–H^δ−^···Y′^δ+^ (hydridic H-bond), and X′–H^δ−^···H′^δ+^–Z (dihydrogen bond). We realize that any change of existing definition is difficult and cannot be rushed and requires an extended, broad, and in-depth discussion within the scientific community. Despite that, the benefit is obvious: the simplification and clarification of the nomenclature of one of the most important type of non-covalent interactions.

Our proposed definition of the hydrogen bond is as follows: The hydrogen bond is an attractive interaction between a hydrogen atom from a molecule or a molecular fragment X–H in which X is more or less electronegative than H and an atom or a group of atoms in the same or a different molecule in which there is evidence of bond formation. A typical hydrogen bond may be depicted as X–H···Y–Z, where the three dots denote the bond.

*Depending on the electronegativity of X, the hydrogen carries a positive charge (protonic hydrogen) and thus acts as the hydrogen-bond donor (Lewis acid) or a negative charge (hydridic hydrogen) and thus acts as the hydrogen-bond acceptor (Lewis base). The atom Y in the molecule in the former case may be an electron-rich region such as, but not limited to, a lone electron pair of Y or a π-bonded pair of Y-Z, while in the second case, it may be an electron-deficient region such as σ-, n-, or π-hole or a positively-charged atom including hydrogen or an ion or a fragment of a molecule.*X–H represents the hydrogen bond donor. The accep-tor may be an atom or an anion Y, or a fragment or a mole-cule Y–Z, where Y is bonded to Z. In some cases, X and Y are the same. In more specific cases, X and Y are the same and X–H and Y–H distances are the same as well leading to symmetric hydrogen bonds. In any event, the acceptor is an electron rich region such as, but not lim-ited to, a lone pair of Y or π-bonded pair of Y–Z.

The other parts of the original IUPAC definition remain unchanged.

## Supporting Information Available

BeH_2_ complexes, figures, and MP2-optimized geometries (PDF)

## References

[ref1] Kollman P. A.; Allen L. C. The Theory of Hydrogen Bond. Chem. Rev. 1972, 72, 283–303. 10.1021/cr60277a004.

[ref2] Reed A. E.; Weinhold F.; Curtiss L. A.; Pochatko D. J. Natural Bond Orbital Analysis of Molecular Interactions: Theoretical Studies of Binary Complexes of HF, H_2_O, NH_3_, N_2_, O_2_, F_2_, CO, and CO_2_ with HF, H_2_O, and NH_3_. J. Chem. Phys. 1998, 84, 5687 10.1063/1.449928.

[ref3] Grabowski S. J. What Is the Covalency of Hydrogen Bonding?. Chem. Rev. 2011, 111, 2597–2625. 10.1021/cr800346f. 21322583

[ref4] Hobza P.; Havlas Z. Blue-Shifting Hydrogen Bonds. Chem. Rev. 2000, 100, 4253–4264. 10.1021/cr990050q. 11749346

[ref5] Hobza P.; Špirko V.; Havlas Z.; Buchhold K.; Reimann B.; Barth H. D.; Brutschy B. Anti-Hydrogen Bond between Chloroform and Fluorobenzene. Chem. Phys. Lett. 1999, 299, 180–186. 10.1016/S0009-2614(98)01264-0.

[ref6] Arunan E.; et al. Defining the Hydrogen Bond: An Account (IUPAC Technical Report). Pure Appl. Chem. 2011, 83, 1619–1636. 10.1351/PAC-REP-10-01-01.

[ref7] Jabłoński M. Binding of X–H to the Lone-Pair Vacancy: Charge-Inverted Hydrogen Bond. Chem. Phys. Lett. 2009, 477, 374–376. 10.1016/j.cplett.2009.07.009.

[ref8] Jabłoński Full vs. Constrain Geometry Optimization in the Open-Closed Method in Estimating the Energy of Intramolecular Charge-Inverted Hydrogen Bonds. Chem. Phys. 2010, 376, 76–83. 10.1016/j.chemphys.2010.08.005.

[ref9] Jabłoński M. Intramolecular Charge-Inverted Hydrogen Bond. J. Mol. Struct. THEOCHEM 2010, 948, 21–24. 10.1016/j.theochem.2010.02.013.

[ref10] Jabłoński M.; Sokalski W. A. Physical Nature of Interactions in Charge-Inverted Hydrogen Bonds. Chem. Phys. Lett. 2012, 552, 156–161. 10.1016/j.cplett.2012.09.061.

[ref11] Jabłoński M. Theoretical Insight into the Nature of the Intermolecular Charge-Inverted Hydrogen Bond. Comput. Theor. Chem. 2012, 998, 39–45. 10.1016/j.comptc.2012.05.023.

[ref12] Jabłoński Charge-Inverted Hydrogen Bond vs. Other Interactions Possessing a Hydridic Hydrogen Atom. Chem. Phys. 2014, 433, 76–84. 10.1016/j.chemphys.2014.01.021.

[ref13] Jabłoński M. Comparative Study of Geometric and QTAIM-Based Differences between X-H···Y Intramolecular Charge-Inverted Hydrogen Bonds, M1···(H–X) Agostic Bonds and M2···(H2-XH) σ Interactions (X = Si, Ge). Comput. Theor. Chem. 2016, 1096, 54–65. 10.1016/j.comptc.2016.09.023.

[ref14] Jabłoński M. Strength of Si-H···B Charge-Inverted Hydrogen Bonds in 1-Silacyclopent-2-Enes and 1-Silacyclohex-2-Enes. Struct. Chem. 2017, 28, 1697–1706. 10.1007/s11224-017-0939-6.

[ref15] Jabłoński M. Ten Years of Charge-Inverted Hydrogen Bonds. Struct. Chem. 2020, 31, 61–80. 10.1007/s11224-019-01454-2.

[ref16] Crabtree R. H.; Siegbahn P. E. M.; Eisenstein O.; Rheingold A. L.; Koetzle T. F. A New Intermolecular Interaction: Unconventional Hydrogen Bonds with Element-Hydride Bonds as Proton Acceptor. Acc. Chem. Res. 1996, 29, 348–354. 10.1021/ar950150s. 19904922

[ref17] Guillot B. A Reappraisal of What We Have Learnt during Three Decades of Computer Simulations on Water. J. Mol. Liq. 2002, 101, 219–260. 10.1016/S0167-7322(02)00094-6.

[ref18] Shea J. A.; Flygare W. H. The Rotational Spectrum and Molecular Structure of the Ethylene–HF Complex. J. Chem. Phys. 1998, 76, 4857.

[ref19] Novick S. E.; Davies P. B.; Dyke T. R.; Klemperer W. Polarity of van Der Waals Molecules. J. Am. Chem. Soc. 1973, 95, 8547–8550. 10.1021/ja00807a008.

[ref20] Bondybey V. E.; Smith A. M.; Agreiter J. New Developments in Matrix Isolation Spectroscopy. Chem. Rev. 1996, 96, 2113–2134. 10.1021/cr940262h. 11848824

[ref21] Potapov A. Weakly Bound Molecular Complexes in the Laboratory and in the Interstellar Medium: A Lost Interest?. Mol. Astrophys. 2017, 6, 16–21. 10.1016/j.molap.2017.01.001.

[ref22] Whittle E.; Dows D. A.; Pimentel G. C. Matrix Isolation Method for the Experimental Study of Unstable Species. J Chem Phys 1954, 22, 1943 10.1063/1.1739957.

[ref23] Jacox M. E. The Spectroscopy of Molecular Reaction Intermediates Trapped in the Solid Rare Gases. Chem. Soc. Rev. 2002, 31, 108–115. 10.1039/b102907j. 12109204

[ref24] Klemperer W.; Vaida V. Molecular Complexes in Close and Far Away. Proc. Natl. Acad. Sci. U. S. A. 2006, 103, 10584–10588. 10.1073/pnas.0508231103. 16740667 PMC1502275

[ref25] Peterson K. A.; Dunning T. H. Accurate Correlation Consistent Basis Sets for Molecular Core–Valence Correlation Effects: The Second Row Atoms Al–Ar, and the First Row Atoms B–Ne Revisited. J. Chem. Phys. 2002, 117, 10548 10.1063/1.1520138.

[ref26] Peterson K. A.; Yousaf K. E. Molecular Core-Valence Correlation Effects Involving the Post-d Elements Ga–Rn: Benchmarks and New Pseudopotential-Based Correlation Consistent Basis Sets. J Chem Phys 2010, 133, 174116 10.1063/1.3503659. 21054015

[ref27] Werner H. J.; Adler T. B.; Manby F. R. General Orbital Invariant MP2-F12 Theory. J Chem Phys 2007, 126, 164102 10.1063/1.2712434. 17477584

[ref28] Peterson K. A.; Adler T. B.; Werner H. J. Systematically Convergent Basis Sets for Explicitly Correlated Wavefunctions: The Atoms H, He, B–Ne, and Al–Ar. J Chem Phys 2008, 128, 084102 10.1063/1.2831537. 18315028

[ref29] Werner H. J.; Knowles P. J.; Knizia G.; Manby F. R.; Schütz M. Molpro: A General-Purpose Quantum Chemistry Program Package. Wiley Interdiscip Rev Comput Mol Sci 2012, 2, 242–253. 10.1002/wcms.82.

[ref30] Werner H. J.; Knowles P. J.; Manby F. R.; Black J. A.; Doll K.; Heßelmann A.; Kats D.; Köhn A.; Korona T.; Kreplin D. A.; Ma Q.; Miller T. F.; Mitrushchenkov A.; Peterson K. A.; Polyak I.; Rauhut G.; Sibaev M. The Molpro Quantum Chemistry Package. J. Chem. Phys. 2020, 152, 144107 10.1063/5.0005081. 32295355

[ref31] Řezáč J. Cuby: An Integrative Framework for Computational Chemistry. J. Comput. Chem. 2016, 37, 1230–1237. 10.1002/jcc.24312. 26841135

[ref32] TURBOMOLE V7.5 2020, a development of University of Karlsruhe and Forschungszentrum Karlsruhe GmbH, 1989–2007, TURBOMOLE GmbH, since 2007; available from http://www.turbomole.com.

[ref33] Adler T. B.; Knizia G.; Werner H. J. A Simple and Efficient CCSD(T)-F12 Approximation. J. Chem. Phys. 2007, 127, 221106 10.1063/1.2817618. 18081383

[ref34] Boys S. F.; Bernardi F. The Calculation of Small Molecular Interactions by the Differences of Separate Total Energies. Some Procedures with Reduced Errors. Mol. Phys. 1970, 19, 553–566. 10.1080/00268977000101561.

[ref35] Jeziorski B.; Moszynski R.; Szalewicz K. Perturbation Theory Approach to Intermolecular Potential Energy Surfaces of van Der Waals Complexes. Chem. Rev. 1994, 94, 1887–1930. 10.1021/cr00031a008.

[ref36] Parrish R. M.; Burns L. A.; Smith D. G. A.; Simmonett A. C.; DePrince A. E.; Hohenstein E. G.; Bozkaya U.; Sokolov A. Y.; di Remigio R.; Richard R. M.; Gonthier J. F.; James A. M.; McAlexander H. R.; Kumar A.; Saitow M.; Wang X.; Pritchard B. P.; Verma P.; Schaefer H. F.; Patkowski K.; King R. A.; Valeev E. F.; Evangelista F. A.; Turney J. M.; Crawford T. D.; Sherrill C. D. Psi4 1.1: An Open-Source Electronic Structure Program Emphasizing Automation, Advanced Libraries, and Interoperability. J. Chem. Theory Comput. 2017, 13, 3185–3197. 10.1021/acs.jctc.7b00174. 28489372 PMC7495355

[ref37] Frisch M. J.; Trucks G. W.; Schlegel H. B.; Scuseria G. E.; Robb M. A.; Cheeseman J. R.; Scalmani G.; Barone V.; Petersson G. A.; Nakatsuji H.; Li X.; Caricato M.; Marenich A. V.; Bloino J.; Janesko B. G.; Gomperts R.; Mennucci B.; Hratchian H. P.; Ortiz J. V.; Izmaylov A. F.; Sonnenberg J. L.; Williams; Ding F.; Lipparini F.; Egidi F.; Goings J.; Peng B.; Petrone A.; Henderson T.; Ranasinghe D.; Zakrzewski V. G.; Gao J.; Rega N.; Zheng G.; Liang W.; Hada M.; Ehara M.; Toyota K.; Fukuda R.; Hasegawa J.; Ishida M.; Nakajima T.; Honda Y.; Kitao O.; Nakai H.; Vreven T.; Throssell K.; Montgomery J. A. Jr.; Peralta J. E.; Ogliaro F.; Bearpark M. J.; Heyd J. J.; Brothers E. N.; Kudin K. N.; Staroverov V. N.; Keith T. A.; Kobayashi R.; Normand J.; Raghavachari K.; Rendell A. P.; Burant J. C.; Iyengar S. S.; Tomasi J.; Cossi M.; Millam J. M.; Klene M.; Adamo C.; Cammi R.; Ochterski J. W.; Martin R. L.; Morokuma K.; Farkas O.; Foresman J. B.; Fox D. J. G16_C01. 2016, p Gaussian 16, Revision C.01, Gaussian, Inc., Wallin.

[ref38] Mallada B.; Gallardo A.; Lamanec M.; de la Torre B.; Špirko V.; Hobza P.; Jelinek P. Real-Space Imaging of Anisotropic Charge of σ-Hole by Means of Kelvin Probe Force Microscopy. Science 2021, 374, 863–867. 10.1126/science.abk1479. 34762455

[ref39] Lo R.; Manna D.; Lamanec M.; Dračínský M.; Bouř P.; Wu T.; Bastien G.; Kaleta J.; Miriyala V. M.; Špirko V.; Mašínová A.; Nachtigallová D.; Hobza P. The Stability of Covalent Dative Bond Significantly Increases with Increasing Solvent Polarity. Nat. Commun. 2022, 13, 1–7. 35440662 10.1038/s41467-022-29806-3 PMC9018688

[ref40] Hougen J. T.; Bunker P. R.; Johns J. W. C. The Vibration-Rotation Problem in Triatomic Molecules Allowing for a Large-Amplitude Bending Vibration. J. Mol. Spectrosc. 1970, 34, 136–172. 10.1016/0022-2852(70)90080-9.

[ref41] Van Der Batsanov S. S. Waals Radii of Elements. Inorg. Mater. 2001, 37, 871–885. 10.1023/A:1011625728803.

