# Supplementary material for: Hydrogen Bonding with Hydridic Hydrogen–Experimental Low-Temperature IR and Computational Study: Is a Revised Definition of Hydrogen Bonding Appropriate?
Source: J Am Chem Soc. 2023 Apr 10;145(15):8550–9. doi: 10.1021/jacs.3c00802 (PMC10119939; doi:10.1021/jacs.3c00802)
Supplement: Supplementary file 1 — ja3c00802_si_001.pdf [file ja3c00802_si_001.pdf]

Hydrogen Bonding with Hydridic Hydrogen – Experimental Low-Temperature IR and Computational Study: Is A Revised Definition of Hydrogen Bonding Appropriate? Supporting Information

Svatopluk Civiš,[*,†,‡] Maximilián Lamanec,[†,¶,§] Vladimír Špirko,[†] Jiří Kubišta,[‡] Matej Špeťko,[¶] and Pavel Hobza[*,†,¶]

†*Institute of Organic Chemistry and Biochemistry, Czech Academy of Sciences, Flemingovo Náměstí 542/2, 16000 Prague, Czech Republic.*

‡*J. Heyrovský Institute of Physical Chemistry, Czech Academy of Sciences, Dolejškova 2155/3, 18200, Prague 8, Czech Republic*

¶*IT4Innovations, VŠB-Technical University of Ostrava, 17. listopadu 2172/15, 70800 Ostrava-Poruba, Czech Republic*

§*Department of Physical Chemistry, Palacký University Olomouc, tr. 17. listopadu 12, 771 46 Olomouc, Czech Republic*

E-mail: svatopluk.civis@jh-inst.cas.cz; pavel.hobza@uochb.cas.cz

Results

Tables

Table S1: Intermolecular distances and changes in intramolecular bond lengths (in Å) of selected bonds in HBeH$\cdots$Y'complexes (Y'= ICF_3, BrCN and HCN) calculated at the MP2-F12/cc-pVTZ-F12 level.

	Be-H closer	Be-H farther	I-C	H$\cdots$I/vdW*
ICF_3	0.003	-0.002	-0.002	2.859/+0.567
	Be-H closer	Be-H farther	Br-C	H$\cdots$Br/vdW
BrCN	0.003	-0.003	-0.003	2.687/+0.613
	Be-H closer	Be-H farther	H-C	H$\cdots$ H/vdW
HCN	0.003	0.015	0.015	2.107/+0.293

* The difference between the intermolecular distance and the sum of vdW radii.

Table S2: The energy characteristics in HBeH$\cdots$Y' complexes (Y'= ICF_3, BrCN and HCN). The total interaction energy calculated at the MP2/cc-pwCVTZ level (ΔE^{MP2}) and at the MP2-F12/cc-pVTZ-F12 level ($\Delta E^{MP2\text{-}F12}$), intrinsic interaction energy with counterpoise correction at the CCSD(T)-F12/cc-pVTZ-F12 level on MP2-F12/cc-pVTZ-F12 geometries ($\Delta E^{CCSD(T)\text{-}F12}$) and Gibbs free energy at MP2/cc-pwCVTZ (ΔG^{MP2}) and MP2-F12/cc-pVTZ-F12 ($\Delta G^{MP2\text{-}F12}$) levels. All values are in kcal/mol.

	$\Delta E^{MP2}/\Delta E^{MP2\text{-}F12}/\Delta E^{CCSD(T)\text{-}F12}$	$\Delta G^{MP2}(18K)/\Delta G^{MP2\text{-}F12}(18K)$
ICF_3	-1.66/-2.36/-2.00	-1.05/-1.94
BrCN	-2.12/-2.41/-2.29	-1.22/-1.49
HCN	-0.92/-1.41/-2.05	-0.18/-0.63

Table S3: The orbital-occupation difference between monomers and in HBeH$\cdots$Y' complexes (Y'= ICF_3, BrCN and HCN) using NBO analysis calculated at the ωB97X-D/aug-cc-pwCVTZ level on MP2-F12/cc-pVTZ-F12 geometries

	σ/σ* Be-H closer	σ/ σ* I-C	ΣLP I
ICF_3	-0.001/0.001	-0.003/0.002	-0.004
	σ/σ* Be-H closer	σ/ σ* Br-C	ΣLP Br
BrCN	0.001/0.001	-0.001/0.008	-0.002
	σ/σ* Be-H closer	σ/ σ* H-C	LP N
HCN	0.003/0.002	-0.001/0.005	-0.001

Table S4: The calculated IR shift calculated at the MP2/cc-pwCVTZ level ($\Delta\nu^{MP2}$) and at the MP2-F12/cc-pVTZ-F12 level ($\Delta\nu^{MP2\text{-}F12}$) in cm^{-1} and the change of intensity calculated at MP2/cc-pwCVTZ level (ΔI in km/mol) of the Be-H band upon complexation

	$\Delta\nu^{MP2}$ / $\Delta\nu^{MP2\text{-}F12}$	ΔI
ICF_3	+1/-1	+167
BrCN	+6/+6	+158
HCN	-11/-35	+82

Figures

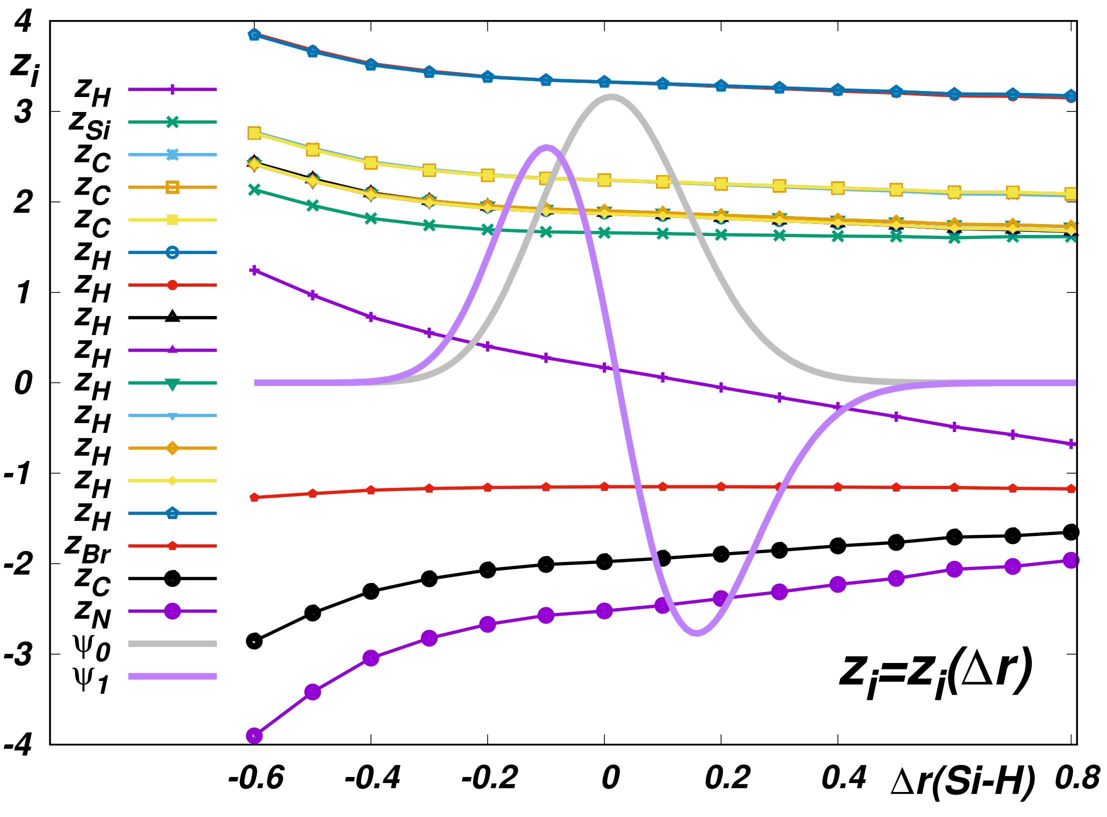

Figure S1: The correlation between the Δr(Si-H) stretch, energy minimum pass and Cartesian atomic coordinates z_i of isolated Me_3Si-H$\cdots$BrCN. ψ_0 and ψ_1 being the wavefunctions of the ground and firstly excited states of the Si-H vibration, respectively.

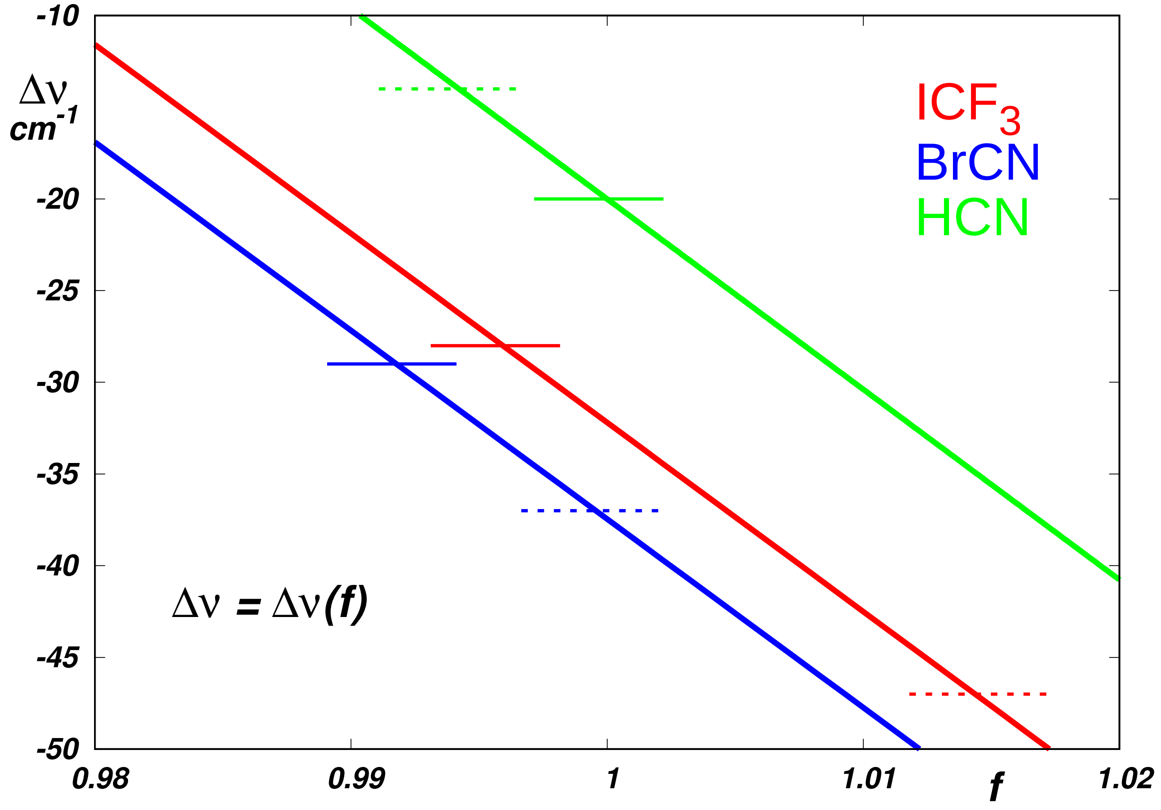

Figure S2: The correlation between the spectral shifts $\Delta\nu$ of the Me_3Si-H$\cdots$Y (Y=ICF_3, BrCN and HCN) and the linear scaling $\mu_{ss} \rightarrow f\mu_{ss}$ of the effective reduced stretching mass μ_{ss} of the probed complexes (slanted lines). Solid and dashed horizontal lines correspond to the spectral shifts $\Delta\nu$ obtained using the deposition on cold substrate and argon matrix isolation techniques, respectively. As it is seen, the dependences are nearly paralel and for instance for $\Delta\nu$(Y=HCN)=-20 they predict $\Delta\nu$(Y=BrCN)=-37 and $\Delta\nu$(Y=ICF_3)=-32 (all shits given in cm^{-1}).

Geometries

Geometries optimized at RI-MP2-F12/cc-pVTZ-F12 level.

ICF_3 monomer

5

I	−0.1973070241	0.1072630298	−2.2723717860
C	0.3726502228	−0.6942982498	−4.1696449346
F	−0.0315441607	−1.9528559134	−4.2840424268
F	−0.1693370090	0.0100899652	−5.1548086617
F	1.6915929710	−0.6643018318	−4.3107171909

BrCN monomer

3

Br	−2.4271215586	−0.2040997100	−1.4047090231
C	−3.9919389489	−0.2393327857	−2.2551114197
N	−5.0212649179	−0.2625088752	−2.8145004487

HCN monomer

3

H	−2.8508092613	−0.2182552853	−1.6324877251
C	−3.7854149837	−0.2418199751	−2.1411847832
N	−4.8063681226	−0.2675616986	−2.6968791308

Me_3SiH monomer

14

C	1.5109832737	0.6037440697	1.3959548514
Si	−0.3511258680	0.4443393817	1.4748616601
C	−0.8434990895	−1.3594214653	1.4200556694
C	−0.9972403123	1.2384608661	3.0403989396
H	−0.9426605562	1.1394147377	0.3043305819
H	1.8183549559	1.6478660422	1.4160102277
H	1.9758939649	0.1023799314	2.2438828422
H	1.9066038844	0.1564235918	0.4857619131
H	−0.7300119517	2.2927344690	3.0868014953
H	−2.0817449523	1.1657211779	3.1006062351
H	−0.5803546343	0.7492212014	3.9198246342
H	−0.4855447588	−1.8381927757	0.5102696358
H	−0.4236816745	−1.8983489158	2.2684686908
H	−1.9255452813	−1.4738013120	1.4542866234

$Me_3SiH \cdots ICF_3$

19

C	1.5681489390	0.5452665558	1.4356164704
Si	−0.2937121148	0.4126579653	1.5183390839
C	−0.9542511915	1.3080357648	3.0193561706
C	−0.8232062569	−1.3790952915	1.5311335247
H	−0.8646727258	1.0617920068	0.3022748252
H	1.8887333731	1.5849031404	1.3961730676
H	2.0222856199	0.0883144593	2.3141898648
H	1.9608514633	0.0394169030	0.5550614738
H	−0.6693275879	2.3584608949	3.0085120163
H	−2.0404977583	1.2550246018	3.0634201629
H	−0.5602749063	0.8639086381	3.9326700853
H	−0.4557862674	−1.9052633109	0.6515743059
H	−0.4313125852	−1.8861952397	2.4121616874
H	−1.9079037919	−1.4703773752	1.5478387675
I	−0.1907403078	0.0958877479	−2.3198194849
C	0.3528753386	−0.6734988552	−4.2397556555
F	−0.0517299955	−1.9320866923	−4.3735099291
F	−0.2016179034	0.0458228124	−5.2092558022
F	1.6713306591	−0.6425707256	−4.4022566340

$Me_3SiH\cdots BrCN$

17

H	−0.0282219156	−0.1456338424	−0.0359143147
Si	−0.0266471467	−0.0626341577	1.4542758106
C	1.7486239672	−0.0459431506	2.0328456135
C	−0.9034894528	1.5104283353	1.9514037163
C	−0.9302427259	−1.5552315180	2.1222585498
H	2.2718021934	−0.9494979729	1.7255347430
H	1.7946780904	0.0141967396	3.1194616529
H	2.2872133802	0.8086012908	1.6275732434
H	−0.4063427929	2.3872818426	1.5402690363
H	−1.9355489597	1.5160678874	1.6042882262
H	−0.9174975362	1.6124720137	3.0359216850
H	−1.9627096597	−1.5812604738	1.7772776505
H	−0.4490522291	−2.4808623199	1.8112540284
H	−0.9445870727	−1.5360767119	3.2113903533
Br	−2.3881148470	−0.2012190260	−1.3578123364
C	−3.9733331309	−0.2347652031	−2.1802716885
N	−5.0145451617	−0.2566484031	−2.7177319700

$Me_3SiH\cdots HCN$

17

H	0.0027368334	−0.0038500060	0.0092597449
Si	0.0001853243	0.0004136004	1.4985058082
C	1.7679213872	0.0016591909	2.1023107246
H	2.2896461431	−0.8971564132	1.7782104187
H	2.3146943017	0.8672159373	1.7319764610
H	1.7962985880	0.0306352272	3.1910117100
C	−0.8864559505	1.5453340362	2.0710284340
H	−0.3868754178	2.4435275524	1.7122078242
H	−0.9122594759	1.5900851590	3.1591099852
H	−1.9139406852	1.5671990527	1.7120304762
C	−0.8928214683	−1.5253007010	2.1019981610
H	−0.9324082786	−1.5352706893	3.1906913217
H	−1.9158956258	−1.5607132666	1.7313637437
H	−0.3798619282	−2.4291590666	1.7779472148
N	1.7731708412	−3.0870353564	−0.0036572690
C	1.4024244912	−2.4415205548	−0.8976750691
H	1.0588349203	−1.8432677024	−1.7084506896

Geometries optimized at RI-MP2/cc-pwCVTZ level

BF_3 monomer

4

B	−0.0005353789	0.0000017799	−2.3343679274
F	0.6560326752	1.1372518931	−2.3352405129
F	0.6560663645	−1.1372291361	−2.3352203492
F	−1.3137066038	−0.0000179229	−2.3326417474

BrCN monomer

3

Br	−2.4265299048	−0.2075410240	−1.4014055510
C	−3.9928843792	−0.2470532767	−2.2541289432
N	−5.0231780836	−0.2730426582	−2.8150171448

COF_2 monomer

4

C	0.3478283669	0.4768401343	−2.2657063902
F	0.8504266080	1.5055712109	−1.6220988626
O	0.9323396357	−0.4880988568	−2.5962789267
F	−0.9259032666	0.7211328987	−2.4731998545

HCN monomer

3

H	−2.8498362182	−0.2182307180	−1.6319578047
C	−3.7850199833	−0.2418100799	−2.1409703683
N	−4.8077361661	−0.2675961610	−2.6976234660

$C_6(CN)_6$ monomer

18

C	−0.5081086372	3.4251599393	3.7266142244
C	−0.6248469708	3.6698403617	2.3490637278
C	−1.5844336344	2.9788377510	1.5921948565
C	−2.4272817553	2.0431545266	2.2128763976
C	−2.3105432683	1.7984739666	3.5904268057
C	−1.3509566237	2.4894765226	4.3472957070
C	0.2297193212	4.6185334978	1.7197523086
C	−3.1651108928	0.8497819597	4.2197380196
C	−1.2325956984	2.2413942872	5.7439992902
C	−1.7027960392	3.2269207910	0.1954915534
C	−3.4002108012	1.3425451395	1.4454844843
C	0.4648194797	4.1257704967	4.4940065499
N	−3.8697098899	0.0675779472	4.7386079061
N	−1.1350075978	2.0368468587	6.8955919421
N	1.2670044942	4.7034310087	5.1267278407
N	0.9343150488	5.4007376155	1.2008782151
N	−1.8003888694	3.4314658559	−0.9561011551
N	−4.2024003497	0.7648894068	0.8127644343

$C_6H_3(CN)_3$ monomer

15

C	0.3231241993	−0.2985873165	1.8283861537
C	−0.6530306613	−1.1396018975	2.3631960312
C	−1.7189398591	−1.5866236897	1.5820337146
C	−1.8009494686	−1.1817237736	0.2495413280
C	−0.8349360501	−0.3410843898	−0.3039466685
C	0.2232281630	0.0950306889	0.4937359763
C	−0.5597317226	−1.5491554245	3.7311446987
N	−0.4833860881	−1.8842857981	4.8505063676
H	−2.4709971649	−2.2378073489	2.0034505971
C	−2.8903635680	−1.6346766688	−0.5602321076
N	−3.7818047790	−2.0053220615	−1.2228527755
H	−0.9053776870	−0.0318726986	−1.3367334996
C	1.2193467502	0.9575320876	−0.0644409651
N	2.0344514016	1.6632942282	−0.5211857226
H	1.1456235346	0.0433840629	2.4397558717

ICF_3 monomer

5

I	−0.1843894617	0.0846604069	−2.3822258377
C	0.3533971079	−0.6774241632	−4.3106019132
F	−0.0550313046	−1.9327030723	−4.4434640694
F	−0.2054869209	0.0500936071	−5.2690161737
F	1.6699158440	−0.6445148647	−4.4714953367

ICN monomer

3

I	1.1474272745	−1.9720083542	−1.4892124408
C	1.9882324471	−3.4167906491	−2.5645252785
N	2.4850000893	−4.2704023337	−3.1998487977

NO_2F monomer

4

N	0.2723602847	0.5184910046	−2.3711489810
F	0.9451984417	1.5862438150	−1.5703549061
O	1.0193338725	−0.3528651260	−2.6407070829
O	−0.8623168609	0.7745853263	−2.5630241721

PCl_3 monomer

4

P	−0.6802432657	−1.1314338479	−2.1758613632
Cl	−2.4569324910	−0.9032636327	−1.2039586863
Cl	−0.0077332851	−2.8486468262	−1.3085724286
Cl	−1.3333582662	−1.8575067952	−3.9645873029

$P(CN)_3$ monomer

7

P	−0.6207902723	−1.0796718141	−2.2000670190
C	−2.0756745166	−0.8170572859	−1.2248956204
N	−2.9972235977	−0.5080155654	−0.5714772533
C	−0.0045129640	−2.4947973233	−1.3315996281
N	0.5274295076	−3.3631545549	−0.7530599938
C	−1.3876331885	−1.9408413717	−3.5443026698
N	−1.8263303229	−2.4204453730	−4.5185924064

$S(CN)_2$ monomer

5

S	−1.4642195877	−1.4567934117	−1.6894049747
C	−2.5620584408	−2.2803414048	−2.6827694628
N	−3.2555239708	−2.8911405772	−3.4059620850
C	−2.5872982435	−0.5239502959	−0.8299002575
N	−3.2993197571	0.1560166897	−0.1914272199

Me_3SiH monomer

14

C	1.5523823990	0.5635193890	1.3848211748
Si	−0.3143488152	0.4364942187	1.4875346755
C	−0.9343492093	1.2842814905	3.0393810236
C	−0.8400179755	−1.3621163539	1.4809906659
H	−0.9048329353	1.1111101746	0.3061850518
H	1.8767155671	1.6031438518	1.3746234403
H	2.0198057844	0.0764516061	2.2402635315
H	1.9307693520	0.0867579125	0.4816950240
H	−0.6490192026	2.3352109765	3.0551619376
H	−2.0198639441	1.2318439517	3.1102547756
H	−0.5185919203	0.8121658451	3.9291866042
H	−0.4991748388	−1.8690738505	0.5793615410
H	−0.4222838274	−1.8892006972	2.3384179501
H	−1.9240514342	−1.4560835151	1.5274306042

$Me_3SiH\cdots BF_3$

18

C	1.8191831312	0.0228999758	2.4541685168
Si	0.2909785775	0.1111652203	1.3779161264
C	−0.7702535514	1.5803502446	1.8396665991
C	−0.6895917028	−1.4780638207	1.4871195480
B	−0.5218425424	−0.1880611061	−2.0476912326
H	0.7406339080	0.2913270910	−0.0300496495
H	2.4508105761	−0.8181768321	2.1722886979
H	2.4132443948	0.9317153715	2.3711394458
H	1.5437628438	−0.1020973950	3.5010830288
H	−1.6392643059	1.6516971071	1.1874030355
H	−0.2116415469	2.5117368570	1.7598036181
H	−1.1263670948	1.4908024059	2.8656604522
H	−1.5867543925	−1.4304521759	0.8722809088
H	−0.0989707017	−2.3300222887	1.1537576613
H	−0.9975624022	−1.6650969806	2.5156692206
F	0.2240757669	0.6607361714	−2.7167726547
F	−0.1971860810	−1.4611069902	−2.0019698315
F	−1.6431198762	0.2200481436	−1.4890564902

$Me_3SiH\cdots BrCN$

17

H	−0.0586806400	−0.1439547640	−0.0172937772
Si	−0.0305280918	−0.0615160528	1.4701555561
C	1.7578988936	−0.0469172014	2.0180883345
C	−0.8982986190	1.5157087221	1.9823758296
C	−0.9254071296	−1.5567459750	2.1531566815
H	2.2746767877	−0.9513153537	1.7007859278
H	1.8247401985	0.0128324286	3.1040970470
H	2.2903596065	0.8074139890	1.6030378355
H	−0.4080307807	2.3915857587	1.5597907438
H	−1.9369776885	1.5216451705	1.6545169735
H	−0.8920467429	1.6209896972	3.0671017539
H	−1.9644780839	−1.5804318927	1.8273404653
H	−0.4513348830	−2.4825624836	1.8303182317
H	−0.9195824017	−1.5415153416	3.2428732882
Br	−2.4228344865	−0.2039778034	−1.4020081609
C	−3.9929713664	−0.2394199588	−2.2566070887
N	−5.0245195724	−0.2625436087	−2.8157056420

$Me_3SiH\cdots COF_2$

18

C	1.7114040717	−0.1708358083	1.6225886333
Si	−0.1222895343	−0.3549278421	1.3006035689
C	−0.9783629326	1.3069599395	1.3963962998
C	−0.8905302764	−1.5602964669	2.5108059551
C	0.2982420018	0.7018948725	−2.1360326141
H	−0.3062402650	−0.9004468436	−0.0708155133
H	2.2227716401	−1.1300209614	1.5542710493
H	2.1645605917	0.5064282292	0.9003300092
H	1.8884900044	0.2316525665	2.6197206642
H	−2.0378022149	1.2206937618	1.1587501696
H	−0.5316016942	2.0167024393	0.7021314792
H	−0.8948146580	1.7220804460	2.4006357697
H	−1.9548477068	−1.6878698762	2.3185704333
H	−0.4183287966	−2.5394502543	2.4456214244
H	−0.7766261489	−1.2022050444	3.5337027928
O	1.0495688452	1.4007313620	−1.5583110711
F	0.6064524605	−0.3929088724	−2.7881926703
F	−0.9946163861	0.8918443516	−2.2554843810

$Me_3SiH\cdots HCN$

17

H	0.0007148243	0.0000802919	0.0217363560
Si	−0.0022960665	0.0048783033	1.5089758916
C	1.7686582223	0.0070945640	2.1138215294
H	2.2889587024	−0.8940935553	1.7933105901
H	2.3163612539	0.8705085079	1.7384473546
H	1.7986368656	0.0417170540	3.2027780724
C	−0.8910790551	1.5532450379	2.0804066170
H	−0.3911612282	2.4513964499	1.7204088024
H	−0.9184359048	1.6005749480	3.1687869598
H	−1.9186687052	1.5746849261	1.7201397610
C	−0.8976654778	−1.5232408165	2.1133647081
H	−0.9429802272	−1.5318110221	3.2023078055
H	−1.9193568080	−1.5605939896	1.7377059453
H	−0.3818807649	−2.4270268075	1.7928703255
N	1.7639822636	−3.0722284220	−0.0148646662
C	1.4165110997	−2.4663933496	−0.9476441732
H	1.0950950059	−1.9059961204	−1.7946828793

$Me_3SiH \cdots C_6(CN)_6$

32

Si	0.3286251471	−0.3036399753	1.8279841167
C	−0.9748167001	−1.4331895899	2.5582525267
C	−0.0566269712	0.0595479927	0.0313727722
C	2.0367564924	−1.0354059912	2.0163775134
H	0.2966429217	0.9857668938	2.5780722982
H	−1.9873619418	−1.0791281834	2.3656200002
H	−0.8968877548	−2.4256624113	2.1129909443
H	−0.8553580916	−1.5470652190	3.6354070328
H	−1.0858023929	0.3865504925	−0.1155349900
H	0.6030758618	0.8240454462	−0.3783049316
H	0.0766568433	−0.8429929204	−0.5661124012
H	2.7975300997	−0.3673707387	1.6153905286
H	2.2714689612	−1.2226126664	3.0631352588
H	2.1081119968	−1.9827059536	1.4826967275
C	−0.5062541945	3.4146790642	3.7211039082
C	−0.6233371266	3.6598145577	2.3443399065
C	−1.5817183734	2.9688367412	1.5874204053
C	−2.4222356005	2.0317206716	2.2079547784
C	−2.3077386077	1.7885629388	3.5854464567
C	−1.3491137486	2.4799402845	4.3416922329
C	0.2397906079	4.5977204080	1.7114552958
C	−3.1609427859	0.8389152215	4.2136972310
C	−1.2223289190	2.2207952992	5.7352377398
C	−1.6992238592	3.2152053375	0.1910027932
C	−3.3820366475	1.3178848438	1.4375161836
C	0.4746250433	4.1061110558	4.4859675353
N	−3.8673237914	0.0565924229	4.7308425134
N	−1.1174587727	2.0031629644	6.8839215790
N	1.2835314819	4.6765629266	5.1168761293
N	0.9527215295	5.3685930689	1.1867448566
N	−1.7991102660	3.4188569402	−0.9609481596
N	−4.1648994407	0.7189860753	0.7992672169

$Me_3SiH\cdots ICF_3$

19

C	1.5724940135	0.5459650482	1.4621666913
Si	−0.2930538428	0.4129120456	1.5390477191
C	−0.9598723804	1.3158633096	3.0370235292
C	−0.8235755445	−1.3821018251	1.5577376931
H	−0.8582063953	1.0548120889	0.3202135138
H	1.8924060973	1.5860306793	1.4159969431
H	2.0255690727	0.0960157395	2.3454377289
H	1.9685981621	0.0340867438	0.5862399048
H	−0.6751384179	2.3668266547	3.0225473234
H	−2.0467963840	1.2631033575	3.0772638435
H	−0.5695453402	0.8762712678	3.9546111748
H	−0.4508538136	−1.9125841113	0.6826194263
H	−0.4379857024	−1.8865559504	2.4435953979
H	−1.9088804500	−1.4727867932	1.5678010408
I	−0.1852228276	0.0862256225	−2.3793414738
C	0.3539155088	−0.6781377763	−4.3069298974
F	−0.0519372282	−1.9358435155	−4.4413288078
F	−0.2044939865	0.0458438246	−5.2701773963
F	1.6717714591	−0.6455424102	−4.4708003545

$Me_3SiH \cdots ICN$

17

H	0.0343746075	−0.0588779123	0.0254222276
Si	0.0078968101	−0.0134994281	1.5176732254
C	1.7770295679	0.0077861319	2.1247816555
H	2.3130028973	−0.8889699350	1.8167377291
H	2.3170814304	0.8712311506	1.7389769096
H	1.8064589397	0.0549490314	3.2132481611
C	−0.8970512963	1.5422566276	2.0240981691
H	−0.3949768728	2.4312340080	1.6457035169
H	−0.9425966480	1.6204441151	3.1100803929
H	−1.9179716313	1.5453741112	1.6456092500
C	−0.8853163619	−1.5407964693	2.1246301294
H	−0.9409339551	−1.5431283027	3.2130927024
H	−0.3707960092	−2.4500232609	1.8165625825
H	−1.9028283248	−1.5833103708	1.7387538732
I	1.1666080976	−2.0048457078	−1.5545729012
C	1.9935417260	−3.4261141044	−2.6866614858
N	2.4805060219	−4.2630696836	−3.3512371356

$Me_3SiH\cdots K^+$

15

C	1.7866298406	0.0000038282	2.0493318013
Si	−0.0000122559	−0.0000376452	1.5182597892
C	−0.8931936751	−1.5472993335	2.0496044957
C	−0.8932823005	1.5471497674	2.0496712834
H	−0.0001872705	−0.0000095638	0.0050462213
H	2.3153733072	−0.8830163517	1.6938209631
H	2.3153437448	0.8830247276	1.6937787146
H	1.8472556262	0.0000315759	3.1374953108
H	−1.9224026687	1.5635775245	1.6942515455
H	−0.3929748144	2.4466102846	1.6942208777
H	−0.9234774686	1.5994843451	3.1378461575
H	−1.9223650241	−1.5636876721	1.6943305150
H	−0.3929314790	−2.4467089999	1.6939612255
H	−0.9232284389	−1.5997800243	3.1377765583
K	−0.0000601249	0.0000485350	−2.5940974577

$Me_3SiH\cdots NO_2F$

18

C	1.7049737006	−0.1853398537	1.5807906432
Si	−0.1358569809	−0.3592496097	1.2948783261
C	−0.9842929319	1.3063949290	1.3896603885
C	−0.8859740970	−1.5455506175	2.5358193955
N	0.1604449131	0.5501548736	−2.2500010635
H	−0.3509118545	−0.9232246278	−0.0641700274
H	2.2062474157	−1.1504576821	1.5199572930
H	2.1487749131	0.4744595205	0.8375401115
H	1.9041831603	0.2324126676	2.5674176053
H	−2.0502848144	1.2179862642	1.1834129138
H	−0.5563524541	2.0019926064	0.6704703299
H	−0.8723638905	1.7368439842	2.3846686227
H	−1.9546458617	−1.6698705264	2.3667068985
H	−0.4202807957	−2.5280866074	2.4740910255
H	−0.7499715544	−1.1751832844	3.5516513100
F	1.0319505817	1.4999685724	−1.4425685865
O	0.7961704961	−0.3416035256	−2.6777175517
O	−0.9563809455	0.9183789165	−2.2673156363

$Me_3SiH \cdots PCl_3$

18

H	0.5098210692	−0.1370539727	0.0811482608
Si	0.2184572143	−0.0418151172	1.5356903499
C	1.8363804352	0.2354821195	2.4385434854
C	−0.9407766048	1.4020900465	1.8149259641
C	−0.5747248876	−1.6401691824	2.1019837670
H	2.5278812964	−0.5884784936	2.2681677795
H	2.3204445980	1.1530881070	2.1072696740
H	1.6694999499	0.3147166437	3.5125551463
H	−0.4945151838	2.3363439084	1.4766998979
H	−1.1749307276	1.5082696303	2.8740577057
H	−1.8768386127	1.2628380067	1.2764612234
H	−0.7936591316	−1.5978370157	3.1690713175
H	−1.5080092093	−1.8284789975	1.5740173079
H	0.0845733303	−2.4896015420	1.9287995620
P	−0.7762010178	−1.1523423599	−2.2509842002
Cl	−2.4801122894	−0.7014884207	−1.2050055028
Cl	−0.2932762443	−2.9484301871	−1.4025119423
Cl	−1.6055600995	−1.7999035822	−4.0069907702

$Me_3SiH \cdots P(CN)_3$

21

H	0.2479959558	−0.1360850346	0.0281075086
Si	0.0944057137	−0.0567327281	1.5151019278
C	1.8102529190	0.1355762085	2.2333912830
C	−0.9783682804	1.4282333805	1.8852859623
C	−0.7105375609	−1.6335175549	2.1117557460
H	2.4376934379	−0.7177960569	1.9802947798
H	2.2972479503	1.0358743312	1.8619894456
H	1.7631293464	0.2045889342	3.3200057337
H	−0.5315942990	2.3461364405	1.5061004311
H	−1.1126376043	1.5416882645	2.9609598655
H	−1.9639103202	1.3163108563	1.4353394369
H	−0.8176596549	−1.6056474205	3.1964883149
H	−1.7049942389	−1.7582481070	1.6855187429
H	−0.1154410101	−2.5087452257	1.8560054126
P	−0.6070487218	−1.0629982382	−2.1927515073
C	−2.0775862456	−0.8061322047	−1.2261578847
N	−3.0152067703	−0.5094558204	−0.5842467019
C	0.0055940237	−2.4917077726	−1.3305198189
N	0.5281658926	−3.3819167387	−0.7708920938
C	−1.3880875243	−1.9435684820	−3.5330163540
N	−1.8305660087	−2.4282040317	−4.5064102302

$Me_3SiH\cdots S(CN)_2$

19

H	−0.0183803652	−0.0514964763	0.0214151472
Si	0.0017365272	−0.0117700010	1.5127372892
C	1.7933877161	0.0046048814	2.0544593095
H	2.3183519529	−0.8865238963	1.7134947465
H	2.3164201877	0.8744709758	1.6600293372
H	1.8634860239	0.0376561537	3.1414620648
C	−0.8889635129	1.5325837296	2.0706793017
H	−0.4019516917	2.4290147291	1.6895426181
H	−0.8995729644	1.5948930348	3.1587644607
H	−1.9208385935	1.5316077021	1.7226595989
C	−0.8624388272	−1.5473774914	2.1465172643
H	−0.4028105703	−2.4549824232	1.7568621825
H	−1.9162671977	−1.5510692943	1.8720127367
H	−0.8051083681	−1.5892159671	3.2343273384
S	−1.4696515183	−1.4523027439	−1.7503767712
C	−2.5671669773	−2.2332128300	−2.7841450453
N	−3.2543822776	−2.8137718778	−3.5379034558
C	−2.6124854712	−0.5878926845	−0.8474518260
N	−3.3476510724	0.0337654794	−0.1749862975

$Me_3SiH\cdots C_6H_3(CN)_3$

29

C	0.3548308475	−0.3465579529	1.8148879055
C	−0.6197604851	−1.1895454051	2.3485922094
C	−1.6869694847	−1.6344356956	1.5685704337
C	−1.7725227148	−1.2243493282	0.2383481918
C	−0.8078938563	−0.3818843729	−0.3147918868
C	0.2513835865	0.0522419728	0.4825070491
C	−0.5262231661	−1.5993815057	3.7160717852
N	−0.4493214221	−1.9353538078	4.8352497137
H	−2.4390641585	−2.2856969109	1.9900385931
C	−2.8638722818	−1.6727716749	−0.5702613796
N	−3.7579190250	−2.0362710302	−1.2339929591
H	−0.8780050321	−0.0730830134	−1.3481955947
C	1.2440555058	0.9183299188	−0.0746913754
N	2.0540456662	1.6296635829	−0.5328521976
H	1.1774056903	−0.0045867649	2.4263201283
C	−4.0129245556	1.7435894049	−0.0000492058
Si	−2.7514912196	2.4065769602	1.2167310526
C	−3.5855517862	3.4833773978	2.5003492928
C	−1.4266999519	3.3748843220	0.3119496890
H	−2.1119256426	1.2594884172	1.9128246964
H	−0.9676969919	2.7925778849	−0.4860690771
H	−1.8559786362	4.2685241152	−0.1418637591
H	−0.6330596231	3.6954231087	0.9860695244
H	−3.5474544489	1.1653719033	−0.7972947892
H	−4.7445356528	1.1020421350	0.4900629796
H	−4.5549952168	2.5660891340	−0.4674596627
H	−4.3472766599	2.9277533249	3.0454555250
H	−2.8655165688	3.8623922140	3.2242144312
H	−4.0686747157	4.3393036661	2.0298286864
